# *Piper* and *Vismia* Species from Colombian Amazonia Differentially Affect Cell Proliferation of Hepatocarcinoma Cells

**DOI:** 10.3390/nu7010179

**Published:** 2014-12-30

**Authors:** Leandro J. Lizcano, Maite Siles, Jenifer Trepiana, M. Luisa Hernández, Rosaura Navarro, M. Begoña Ruiz-Larrea, José Ignacio Ruiz-Sanz

**Affiliations:** Department of Physiology, Medicine and Dentistry School, University of the Basque Country UPV/EHU, Leioa 48940, Spain; E-Mails: lizcanomvz@gmail.com (L.J.L.); msg_@hotmail.com (M.S.); jtrepiana001@ikasle.ehu.es (J.T.); luisa.hernandez@ehu.es (M.L.H.); rosaura.navarro@ehu.es (R.N.); joseignacio.ruizs@ehu.es (J.I.R.S.)

**Keywords:** antioxidant activity, free radical, polyphenol, hepatoma cell line, cell cycle arrest, flow cytometry, superoxide dismutase, catalase

## Abstract

There is an increasing interest to identify plant-derived natural products with antitumor activities. In this work, we have studied the effects of aqueous leaf extracts from Amazonian *Vismia* and *Piper* species on human hepatocarcinoma cell toxicity. Results showed that, depending on the cell type, the plants displayed differential effects; thus, *Vismia baccifera* induced the selective killing of HepG2, while increasing cell growth of PLC-PRF and SK-HEP-1. In contrast, these two last cell lines were sensitive to the toxicity by *Piper krukoffii* and *Piper putumayoense*, while the *Piperaceae* did not affect HepG2 growth. All the extracts induced cytotoxicity to rat hepatoma McA-RH7777, but were innocuous (*V. baccifera* at concentrations < 75 µg/mL) or even protected cells from basal death (*P. putumayoense*) in primary cultures of rat hepatocytes. In every case, cytotoxicity was accompanied by an intracellular accumulation of reactive oxygen species (ROS). These results provide evidence for the anticancer activities of the studied plants on specific cell lines and suggest that cell killing could be mediated by ROS, thus involving mechanisms independent of the plants free radical scavenging activities. Results also support the use of these extracts of the *Vismia* and *Piper* genera with opposite effects as a model system to study the mechanisms of the antitumoral activity against different types of hepatocarcinoma.

## 1. Introduction

Cancer is a major cause of death worldwide and the liver cancer the third most common cause of cancer death [1]. Among the different tumors the incidence of primary liver malignancy has increased dramatically over the past 20 years, with hepatocellular carcinoma being the most common primary liver tumor [2]. Therefore, the development of chemotherapeutic agents is important to reduce the incidence of mortality, and thus requiring better knowledge of the biology of cancer cells, *i.e.*, their differential signaling systems, protein expression, and specific metabolism. Cancer cells have a different genotype and phenotype from noncancerous cells. Thus, they show a high glycolysis rate, this pathway supporting the increase in energy demand to allow cellular function, proliferation, and tumor growth [3,4,5,6,7].

There are also differences in the activity of drug metabolizing enzymes; hepatoma cells have negligible levels on various P450 cytochromes [8,9]. This lack of phase I enzymes makes tumor cells more tolerant to certain concentrations of drugs that generate toxicity in normal hepatocytes after they have been metabolized. These differential characteristics between cancer and normal cells could have clinical applications.

There is an increasing interest in identifying chemotherapeutic agents that can prevent tumor initiation, delay or stop tumor growth and metastasis or reduce mortality. Natural products derived from plants have recently received considerable attention because of their properties, including antioxidant, anti-inflammatory and antitumor activities. Plants play a key role as sources of effective anticancer agents. It is significant that about 60% of the anticancer drugs currently used are derived from natural sources, including plants, marine organisms and microorganisms. Medicine based on plants and their active components has found a role in cancer treatment. Phenolic compounds extracted from medicinal herbs have shown interesting biochemical properties and pharmacological activities, mainly due to their antioxidant potential and the inhibition exerted on key enzymes in the inflammatory response, such as cyclooxygenase [10,11].

Colombia has a great diversity of plant species that are a source of natural products that can be used in treating diseases. Many of these species are used in folk medicine, and have been found to exert antimicrobial [12], antiplasmodial [13], antiprotozoal, anti-inflammatory [14], anti-HIV [15], and anticancer [16] activities. In previous reports we described the content of total phenols and flavonoids, and the *in vitro* antioxidant activities of aqueous extracts from Colombian Amazonian plants prepared as infusions as are commonly used in traditional medicine [17,18]. From the analyzed extracts we have selected, due to their high antioxidant potential, *Vismia baccifera*, *Piper krukoffii* and *Piper putumayoense* species. Aqueous extracts from leaves of these plants from *Hypericaceae* and *Piperaceae* families were tested for their effect on toxicity of hepatocarcinoma cells. An initial approach to the possible mechanism of action involved was also studied.

## 2. Experimental Section

### 2.1. Materials

The human hepatoma cell lines HepG2, PLC/PRF/5 and SK-HEP-1 and the rat hepatoma cell line McA-RH7777 were purchased from American Type Culture Collection (ATCC) (Manassas, VA, USA). Eagle’s Minimum Essential Medium (EMEM), fetal bovine serum (FBS) and horse serum were obtained also from ATCC. l-glutamine, streptomycin-penicillin solution, propidium iodide, Tween-20, glutahione reductase (GR) (EC 1.6.4.2), NaN_3_, trypsin, EDTA-Na_2_, 30% H_2_O_2_ solution, and 3-(4,5-dimethylthiazo-2-yl)-2,5-diphenyl-tetrazolium bromide (MTT) were all obtained from Sigma-Aldrich (St Louis, MO, USA). 2′,7′-dichlorodihydrofluorescein diacetate (H_2_DCF-DA) was obtained from Molecular Probes (Eugene, OR, USA). NADPH was purchased from Calbiochem (Darmstadt, Germany). GSH was from Boehringer Mannheim GmbH (Ingelheim am Rheim, Germany). RNase A was obtained from Roche Biochemicals (Indianapolis, IN, USA). Superoxide dismutase determination kit was purchased from Fluka (Basel, Switzerland).

### 2.2. Preparation and Characterization of the Plant Extracts

The plants were collected from the Macagual Research Centre forest in Florencia, Caquetá (Colombia), and taxonomically identified by botanical experts and deposited at the Herbarium of the Botanical Garden of Amazonia University—HUAZ (Florencia, Colombia). The samples were processed in the laboratory within a maximum of 24 h after harvesting. Otherwise, the material was stored under refrigeration at 4 °C. The plant extracts were prepared as aqueous infusions, as generally used in folk medicine. For this purpose, the leaves of the fresh plants were rinsed in water, cut into tiny pieces and boiled in 500 mL of water with constant shaking for 15 min. The mixture was allowed to settle for 10 min and stored at −20 °C. The samples were carried to the Department of Physiology of the University of the Basque Country (Spain). Once defrosted, samples were centrifuged at 1200 g for 5 min at 4 °C, and the supernatant was sterilized by filtration (0.22 µm pore size). Aliquots were stored at −80 °C until use. Several aliquots of the extracts were dried in a Savant SpeedVac concentrator (Thermo Fisher Scientific, Waltham, MA, USA) to estimate the dry weight. The extracts were characterized in terms of the content of total phenols and flavonoids (by colorimetric assays), and the total antioxidant activity, measured as the Trolox equivalent antioxidant capacity (TEAC) and the oxygen radical absorbance capacity (ORAC), as is described in [17].

The leaf extracts that we have used in this work contained per gram of dry weight: a) 43.2 ± 0.3 mg gallic acid (total phenols) and 23.4 ± 0.2 mg catechin (total flavonoids) for *V. baccifera*; b) 16.8 ± 0.1 mg gallic acid (total phenols) and 8.7 ± 0.1 mg catechin (total flavonoids) in the case of *Piper krukoffii* [17]; and c) 22.2 ± 0.1 mg gallic acid (total phenols), and 10.2 ± 0.1 mg catechin (total flavonoids) for *Piper putumayoense* [17]. The total antioxidant activity of the extracts were: a) 355.3 ± 5.2 µmol Trolox/g (TEAC) and 922.3 ± 19.5 µmol Trolox/g (ORAC) for *V. baccifera*; b) 92.0 ± 3.8 µmol Trolox/g (TEAC) and 247.1 ± 18.5 µmol Trolox/g (ORAC) in the case of *Piper krukoffii* [17]; and c) 91.0 ± 12.3 µmol Trolox/g (TEAC) and 359.1 ± 44.4 µmol Trolox/g (ORAC) for *Piper putumayoense* [17].

### 2.3. Rat Liver Hepatocyte Isolation and Maintenance in Primary Cultures

Hepatocytes were isolated from male Sprague-Dawley rats (180–200 g) by collagenase perfusion, as previously described [19]. The cellular suspension obtained was filtered through a nylon mesh and incubated for 3 min in a syliconized Erlenmeyer for 3 min at 37 °C under a 95% O_2_/5% CO_2_ atmosphere and constant shaking. Cells were centrifuged at 50× *g* for 3 min at room temperature to remove death cells and cellular debris. The hepatocyte viability, determined by the trypan blue exclusion test, was typically greater than 90%. Hepatocyte primary cultures were prepared as previously reported [20].

The experimental use of animals followed the European Directives and Recommendation (2003/65/CE and 2007/526/CE) regarding the welfare of animals used in scientific procedures and the protocol was approved by the Ethical Committee of Animal Welfare of the University of the Basque Country UPV/EHU (ref. CEBA/24-P02/2009).

### 2.4. Culture and Maintenance of Hepatocarcinoma Cell Lines

Human liver cancer HepG2, PLC/PRF/5 and SK-HEP-1 cell lines were maintained in EMEM supplemented with 10% heat inactivated FBS, 2 mM l-glutamine, 0.1 mg/mL streptomycin and 100 U/mL penicillin. The rat hepatoma McA-RH7777 cell line was maintained in EMEM supplemented with 20% heat inactivated horse serum, 5% heat inactivated FBS, 2 mM l-glutamine, 0.1 mg/mL streptomycin and 100 U/mL penicillin. Cells were grown in 75 cm^2^ flasks at 37 °C in humidified atmosphere with 5% CO_2_. Medium was replaced every 2 to 3 days. When the cell monolayer reached 70% of confluence, cells were detached with a solution of 0.1% trypsin-0.04% EDTA and then harvested to perform subsequent experiments.

The cell line culture procedures used in this work were approved by the Ethical Committee of Research involving Biological Agents and Genetically Modified Organisms (CEIAB/ABIEB) of the University of the Basque Country UPV/EHU (refs. CEIAB/121/2012 and CEIAB/122/2012).

### 2.5. Cellular Toxicity

Cell toxicity was assessed by MTT assay based on the enzymatic reduction of the yellow tetrazolium salt into purple formazan by metabolically active cells [21]. Briefly, liver cell lines and primary rat hepatocytes were seeded onto Petri dishes and treated without (control) or with the aqueous plant extracts at different concentrations for 24 and 48 h. After treatments cells were washed and incubated with MTT for 3 h, and the resultant formazan crystals were solubilized with a dimethyl sulfoxide (DMSO):NaOH 10 N solution for 30 min in the dark. Aliquots were taken up and moved into 96-well plates and the absorbance registered at 550 nm in a microplate reader.

The toxic effectiveness of the extracts was measured in terms of LC50, the extract concentration leading to 50% reduction of the formazan absorbance. It was calculated by non-linear regression analysis, fitting the data to polynomial equations, using GraphPad Prism version 4.01 (GraphPad, San Diego, CA, USA).

### 2.6. Cell Cycle Analysis

Cells were seeded at a density of 300,000 cells onto Petri dishes and incubated for 24 h without (control) or with the plant leaf extracts. After treatments, cells were washed with phosphate buffered saline (PBS), trypsinized, harvested and fixed in 70% ice-cold ethanol overnight at 4 °C. The following day, the cells were washed with ice-cold PBS after discarding the ethanol and stained with 25 µg/mL propidium iodide in the presence of 200 µg/mL RNase A for 45 min at 37 °C in the dark. The cell cycle distribution of cells was determined by flow cytometry (Beckman Coulter Gallios) in the General Research Services SGIker of the UPV/EHU (http://www.ikerkuntza.ehu.es/p273-sgikerhm/en/) with a total acquisition of 10,000 events. The percentage of cells in different phases of the cell cycle was analyzed by Summit 4.3 software (Dako, Glostrup, Hovedstaden, Denmark).

### 2.7. Intracellular ROS Detection

Intracellular ROS generation was estimated by using the cell-permeant reagent 2′,7′-dichlorodihydrofluorescein diacetate (H_2_DCF-DA), which is deacetylated and oxidized inside the cell forming the fluorescent compound, 2′,7′-dichlorofluorescein (DCF). Primary rat hepatocytes were seeded at a density of 10,000 cells per well onto 96-well plates 24 h prior the addition of treatments. HepG2, SK-HEP-1 and McA-RH7777 were seeded at a density of 2000 cells per well 48 h before starting the corresponding treatments. The media were renewed and cells were incubated for 24 h in their corresponding medium without (control) and with the plant leaf extracts. The cells were washed and incubated with 10 µM H_2_DCF-DA for 30 min at 37 °C in the dark. Then the probe solution was removed and, after washing twice with PBS, the cells were lysed by the addition of 200 µL of 1% Tween-20 solution. The DCF fluorescence was measured using a 96-well plate reader at an excitation wavelenght of 485 nm and an emission wavelength of 528 nm. Cell fluorescence without the addition of H_2_DCF-DA was used to correct for autofluorescence. Results were expressed as the percentage fluorescence in control cells.

### 2.8. Cell Protein Assay

Cells were harvested and lysed in PBS by two freeze-thaw cycles in liquid nitrogen. Protein was quantified spectrophotometrically at 595 nm by Coomassie Blue dying [22], using bovine serum albumin as standard.

### 2.9. Enzymatic Assays

Superoxide dismutase (SOD), catalase and glutathione peroxidase activities were measured in HepG2, SK-HEP-1 and McA-RH7777 cell lines. Glutathione peroxidase and catalase specific activities were derived from regression lines obtained by plotting the rate of absorbance change *versus* assayed protein amounts. At least four different protein amounts were assayed for each independent experiment.

#### 2.9.1. Superoxide Dismutase

SOD activity was measured using a SOD assay kit, according to manufacturer’s instructions. The assay is based on inhibition of WST-1 (a water soluble tetrazolium salt) reduction with xanthine-xanthine oxidase system used as a superoxide generator. The reaction took place in a final volume of 275 µL and started with the addition of WST-1, which is transformed to chromogenic WST-1 formazan. The increase in absorbance was monitored at 450 nm every 60 s for 15 min in a 96-well plate reader at 37 °C. A calibration curve was obtained assaying known quantities of a SOD commercial source (Sigma-Aldrich, St Louis, MO, USA). The results were expressed as SOD units/mg of protein.

#### 2.9.2. Glutathione Peroxidase

Glutathione peroxidase activity was assayed by the indirect method of Flohé and Güntzler [23], based on a coupled enzyme system where NADPH is consumed by glutahione reductase to convert the generated glutathione disulfide (GSSG) to its reduced form. The reaction mixture contained 50 mM potassium phosphate buffer (pH 7.0), 1 mM EDTA-Na_2_, 0.5 mM NaN_3_, 0.45 mM GSH, 0.2 mM NADPH and 0.45 U of glutahione reductase in a total volume of 225 µL. The reaction started by the addition of cumene hydroperoxide (0.72 mM final concentration). The decrease in absorbance was monitored at 340 nm every 60 s for 15 min in a 96-well plate reader at 30 °C. The results were expressed as nmol/min/mg protein using the experimental extinction coefficient of 3.065 mM^−1^.

#### 2.9.3. Catalase

Catalase activity was measured according to Aebi [24] by spectrophotometric detection of the H_2_O_2_ disappearance at 240 nm. The reaction took place at 25 °C in a final volume of 1 mL containing 90 mM potassium phosphate buffer (pH 6.8) and started with the addition of H_2_O_2_ (30 mM final concentration). Decrease in absorbance was continuously measured every 2 s over 1 min. Catalase activity was expressed as μmol/min/mg of protein using the extinction coefficient at 240 nm ε = 0.04 mM/cm.

### 2.10. Statistical Analysis

Data were expressed as mean ± standard error (SE) from at least three independent experiment**s**. Means of related groups were compared by Paired-Samples Student’s *t*-test using SPSS 17.0 statistical package (SPSS Inc., Chicago, IL, USA). Statistical significance was assumed at *p* < 0.05.

## 3. Results

### 3.1. Cell Toxicity

**Figure 1:**
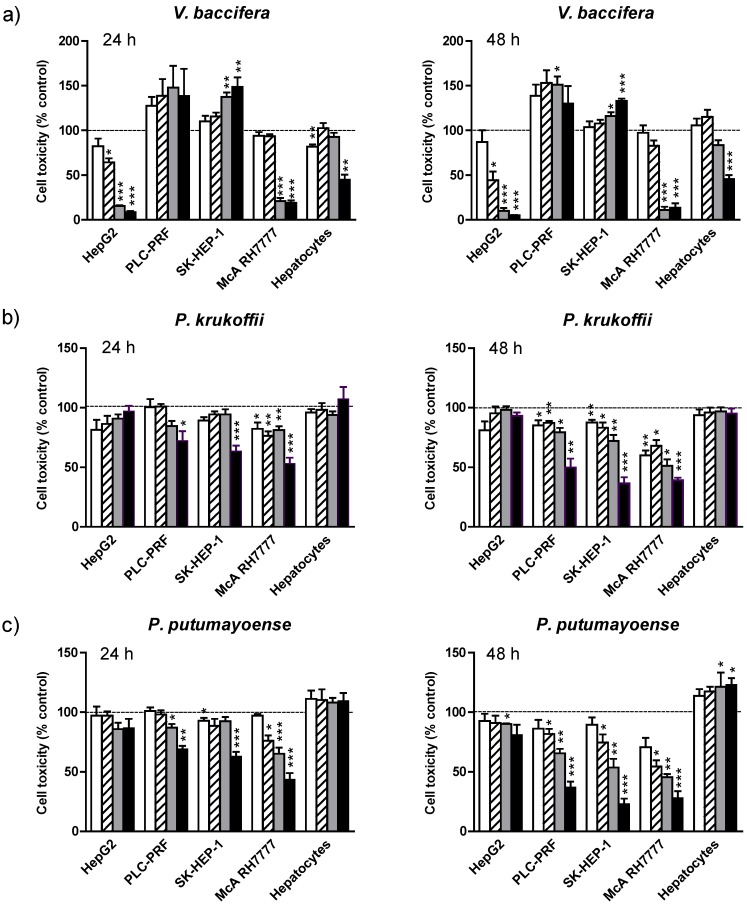
Effects of (**a**) *V. baccifera*; (**b**) *P. krukoffii*; and (**c**) *P. putumayoense* leaf extracts on the cytotoxicity to tumor cell lines and primary hepatocyte cultures. The extracts were assayed at the concentrations: 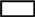 19 µg/mL; 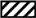 38 µg/mL; 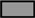 75 µg/mL; 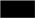 150 µg/mL for 24 h and 48 h. Cell toxicity was determined by the MTT cholorimetric assay, as described in Mat and Met. Results are the means + standard error (SE) of the mean of *n* = 3–6 experiments. * *p* < 0.05; ** *p* < 0.01; *** *p* < 0.005.

The effects of different concentrations of the extracts on MTT in the human hepatoma cell lines HepG2, PLC-PRF, and SK-HEP-1, the rat hepatoma McA-RH7777 cell line and rat hepatocytes are depicted in Figure 1. The *V. baccifera* extract markedly decreased the MTT formazan absorbance in human HepG2 cells in a dose- and time-dependent manner (significant effects were observed at 19 µg/mL, and up to a 95% loss of viability was noted with 150 µg/mL). In the rat hepatoma McA-RH7777 cell line only the highest concentrations produced a significant toxicity (higher than 80%) (Figure 1a).

At 150 µg/mL, the extract strongly decreased cell viability in non-malignant rat hepatocytes (55% reduction at 24 h and 48 h). SK-HEP-1 and PLC-PRF tumor cell lines behaved differently. Thus, the extract increased cellular proliferation, the differences being statistically significant in SK-HEP-1 with the highest doses (up to 37% increase was observed with 75 µg/mL and up to 49% increase with 150 µg/mL by 24 h) and in PLC-PRF with intermediate concentrations (50% increase at 38 µg/mL by 48 h).

*P. krukoffii* displayed cytotoxic activities in PLC-PRF and SK-HEP-1 human cell lines, and the rat hepatoma McA-RH7777 (Figure 1b). The toxicity was dose- and time-dependent. The extract did not modify cell growth of HepG2 or the viability of non-transformed hepatocytes.

The *P. putumayoense* leaf (19 µg/mL–150 µg/mL) was clearly cytotoxic to McA-RH7777, SK-HEP-1, and PLC-PRF hepatoma cell lines, HepG2 being less susceptible to the extract toxicity (Figure 1c). By contrast, the aqueous extract increased the viability of non-transformed rat liver cells by at least 10%.

The LC50 value (concentration that is toxic to 50% of the cells) at 48 h was used as a parameter for cytotoxicity. From the experiments it can be summarized that the rat hepatoma McA-RH7777 cell line is the most susceptible to toxicity, since the two-day exposure to any of the aqueous extracts (at concentrations lower than 80 µg/mL) resulted in cell toxicity by 50% (Table 1). Also, *V. baccifera*, depending on the cell type, displayed differential effects; so, it was highly cytotoxic to human tumor HepG2 (LC50 lower than 40 µg/mL), while increasing human malignant liver PLC-PRF and SK-HEP-1 cell growth. *P. krukoffii* induced toxicity to human PLC-PRF and SK-HEP-1 hepatic cancer cells, but at the highest concentration used it did not affect the viability of HepG2 or nontumoral hepatocytes. Finally, *P. putumayoense* was cytotoxic to human PLC-PRF and SK-HEP-1 hepatoma cells, did not affect HepG2 growth, and even protected non-transformed hepatocytes from basal death.

**Table 1:** Concentration (µg/mL) that causes 50% of cell toxicity (LC50) at 48 h.

Cell type	LC50 (µg/mL)		
	*V. baccifera*	*P. krukoffii*	*P. putumayoense*
HepG2	35	n.d.	n.d.
PLC-PRF	Proliferative	149	106
SK-HEP-1	Proliferative	126	83
McA RH7777	57	76	47
Primary cultured hepatocytes	100	n.d.	Protective against basal cell death

n.d., not detected (assayed at the maximal concentration of 150 µg/mL). Results are the means of *n* = 4. LC50 values were derived by non-linear regression analysis, fitting the data to polynomial equations.

### 3.2. Mechanism of Toxicity

As indicated above, *P. krukoffii* and *P. putumayoense* extracts were particularly interesting, since they induced selective killing in human (*i.e.*, SK-HEP-1) and rat (McA-RH7777) tumor cells, but were innocuous (*P. krukoffii*) or even increased cell viability (*P. putumayoense*) in normal rat hepatocytes. *V. baccifera* leaf in a concentration lower than 100 µg/mL behaved similarly, as it was cytotoxic in human (HepG2) and rat (McA-RH7777) hepatoma cells, but did not affect nontransformed hepatocyte viability. Deregulation of cell cycle progression, an excessive production of ROS or a reduced protective defense in cancer cells are processes often involved in cell death. We then assessed specific parameters that could provide some information on the mechanism involved in cytotoxicity. Thus, we analyzed cell cycle arrest by flow cytometry and ROS generation by the 2′,7′-dichlorofluorescein fluorescent probe. For comparative purposes, ROS generation was also determined in normal rat hepatocytes exposed to the extracts.

#### 3.2.1. Cell Cycle Arrest

Cell cycle was studied in McA-RH7777 cells, which were sensitive to all the extracts. As can be seen in Table 2, *V. baccifera* assayed at the concentration inducing cytotoxicity increased significantly the number of cells in subG0 phase, blocking cell cycle at G2/M. The population of cells in G2/M increased by 55% (*p* < 0.05); this increase in the number of cells in G2/M was accompanied by a 10% reduction in the *S*-phase.

*P. krukoffii* did not alter cell cycle in the rat hepatoma McA-RH7777 cell line. By contrast, *P. putumayoense* dramatically increased the number of cells in subG0 (*p* < 0.005) and induced cell cycle arrest at the G2/M phase.

**Table 2:** Flow cytometry analysis of the effects of plant leaf extracts on rat hepatoma McA-RH7777 cells. Cells in subG_0_ were expressed as the percentage of the total cells (SubG0 + G0/G1 + S + G2/M).

Treatment	SubG0 (%)	Cell cycle		
		G0/G1(%)	S (%)	G2/M (%)
No additions	8.9 ± 3.1	59.8 ± 1.0	27.8 ± 1.9	12.8 ± 1.3
*V. baccifera* (75 µg/mL)	66.1 ± 14.5 *	39.6 ± 7.5 *	27.1 ± 2.0 *	33.3 ± 4.9 *
*P. krukoffii* (150 µg/mL)	9.5 ± 2.3	57.2 ± 2.2	29.0 ± 0.4	13.8 ± 0.7
*P. putumayoense* (150 µg/mL)	16.3 ± 1.4 ***	45.9 ± 1.4 ***	36.2 ± 1.5 *	17.9 ± 1.8 *

Results are the mean ± standard error of the mean of *n* = 3–6 experiments. * *p* < 0.05; ** *p* < 0.01; *** *p* < 0.005, significantly different from control (no additions).

#### 3.2.2. ROS Production

**Figure 2:**
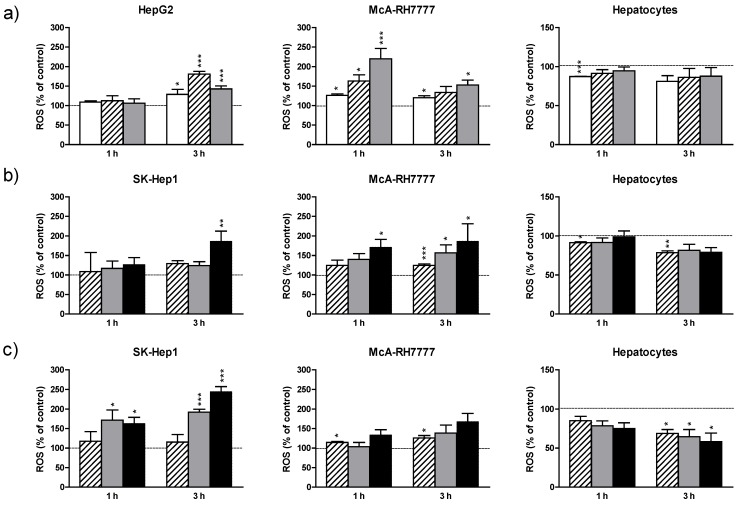
Effects of (**a**) *Vismia*; (**b**) *P. krukoffii*; and (**c**) *P. putumayoense* leaf extracts on ROS formation in hepatoma lines, and primary cultures of rat hepatocytes. The cells were incubated without (control) or with the plant extracts at the concentrations: 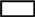 19 µg/mL; 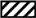 38 µg/mL; 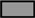 75 µg/mL; 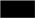 150 µg/mL for 1 and 3 h. After incubation, cells were loaded with the fluorescent probe as described in Mat. and Meth. Results are expressed as the percentage of the control values and are the mean + standard error (SE) of the mean of *n* = 3 experiments. * *p* < 0.05; ** *p* < 0.01; *** *p* < 0.005.

Next, we examined the effects of the plant extracts on the intracellular ROS production. SK-HEP-1 was chosen as the representative cell line of those sensitive to the *Piperaceae* toxicity. Figure 2 shows ROS levels in hepatoma cell lines and normal hepatocytes exposed to the extracts. *V. baccifera* increased ROS formation in the *Hypericaceae*-sensitive cell line HepG2. In McA-RH7777, *V. baccifera* dose-dependently induced the generation of ROS at 1 h and to a lesser extent at 3 h. Similarly to *Vismia*, both *Piperaceae* induced the formation of ROS in McA-RH7777 in a dose- and time-dependent manner. In SK-HEP-1 the *Piper* extracts increased ROS levels. In contrast to tumor cell lines, *Vismia* and *Piper* extracts either maintained constant the basal levels of ROS or even reduced them in normal rat hepatocytes (Figure 2).

### 3.3. Antioxidant Activities

Table 3 shows the antioxidant activities detected in the cell lines after 24 h and 48 h of exposure with the extracts. Under control conditions (no additions) the activity of catalase in HepG2 was about 8-fold higher than in SK-HEP-1, while basal SOD and glutathione peroxidase activities were similar in both human cell lines. The rat hepatoma cell line exhibited higher glutathione peroxidase and catalase basal activities than in the human cell lines.

The *V. baccifera* leaf extract significantly reduced catalase activity in McA-RH7777 cells (19% decrease at 24 h, *p* < 0.05; 33% decrease at 48 h, *p* < 0.01). SOD activity was also decreased at 48 h. The *P. krukoffii* and *P. putumayoense* extracts markedly reduced catalase activity in this rat hepatoma cell line (40% and 23% reduction at 24 h, respectively; 45% reduction at 48 h by both *Piper* species). The *Piper* extracts increased glutathione peroxidase activity by 25% at 24 h. Hydrogen peroxide can be neutralized by the enzymatic scavengers, catalase and glutahione peroxidase. Although both enzymes play similar roles in transforming hydrogen peroxide into water, glutathione peroxidase *per se* is not an efficient decomposer, and high levels of hydrogen peroxide have been detected in glutahtione peroxidase-sufficient but catalase-depleted cells [25]. Catalase exhibits one of the highest turnover rates among all known biological enzymes, and in the studied cells it showed an activity against hydrogen peroxide decomposition three orders of magnitude higher than glutathione peroxidase (Table 3).

**Table 3:** Antioxidant enzyme activities in hepatoma cell lines exposed to *V. baccifera* (75 µg/mL), *P. krukoffii* (150 µg/mL), and *P. putumayoense* (150 µg/mL) leaf extracts for 24 and 48 h. Results are the mean ± SE of *n* = 3–9. * *p* < 0.05; ** *p* < 0.01; *** *p* < 0.005, significantly different from control.

Treatment		24 h			48 h		
		SOD U/mg	GPx nmol/min/mg	Catalase µmol/min/mg	SOD U/mg	GPx nmol/min/mg	Catalase µmol/min/mg
McA-RH7777	Control	22.0 ± 0.9	38.0 ± 4.2	134.0 ± 8.6	23.4 ± 5.2	40.6 ± 3.6	143.7 ± 5.2
	*V. baccifera*	19.3 ± 3.4	44.6 ± 7.4	108.2 ± 8.1 *	14.8 ± 3.2 *	54.6 ± 9.3	95.8 ± 15.8 **
	*P. krukoffii*	22.1 ± 2.3	47.0 ± 1.7 **	80.4 ± 3.6 ***	23.3 ± 1.8	45.8 ± 3.1	77.3 ± 6.6 **
	*P. putumayoense*	21.0 ± 1.7	47.9 ± 3.5 **	103.2 ± 5.9 ***	23.4 ± 1.9	49.5 ± 2.3 *	81.0 ± 7.3 **
HepG2	Control	15.5 ± 1.5	1.86 ± 0.50	104.6 ± 10.1	18.9 ± 1.7	1.84 ± 0.16	101.2 ± 8.0
	*V. baccifera*	21.4 ± 1.8 *	1.57 ± 0.27	65.2 ± 15.5*	20.8 ± 2.8	1.76 ± 0.51	73.0 ± 17.5
SK-HEP-1	Control	16.0 ± 1.0	2.18 ± 0.46	11.6 ± 2.3	17.4 ± 0.7	3.72 ± 0.63	13.6 ± 3.3
	*P. krukoffii*	19.0 ± 1.0 *	2.62 ± 0.69	3.9 ± 3.0	22.9 ± 1.1 ***	2.57 ± 1.33	7.8 ± 1.9
	*P. putumayoense*	19.4 ± 0.4 ***	1.30 ± 0.80	7.6 ± 3.4	25.3 ± 1.1 ***	2.10 ± 0.26*	9.6 ± 5.3

SOD, superoxide dismutase; GPx, glutathione peroxidase.

*V. baccifera* increased SOD activity in HepG2, while reducing catalase activity at 24 h. *P. krukoffii* and *P. putumayoense* significantly increased SOD activity in SK-HEP-1, the hepatoma cell line target of the cytotoxic actions of these *Piperaceae*. In the case of *P. putumayoense*, a decrease was also found in the activity of glutathione peroxidase.

These results on the effects of the plant extracts on antioxidant activities, *i.e.*, a general increase in SOD activity and a reduced catalase activity, suggest that the extracts increase the intracellular hydrogen peroxide levels at long periods.

## 4. Discussion

In this work we have used several human hepatoma cell lines as model system to study the cytotoxic and antiproliferative activities of various plant species from Colombian Amazonia. This system, easy to manipulate, serves as a model for human cancer to study the activities of novel anticancer drugs, the sensitivity patterns, and their mechanisms of action that could lead to the development of new therapeutic targets. The results of the research in cancer cell lines are usually extrapolated to *in vivo* human tumors [26] and have been recognized by pharmaceutical companies as models for the screening and characterization of anticancer therapeutics [27]. The use of cell lines has, however, some limitations; for example, the cell culture environment is different from that of the original tumor, and tumor cell lines have lost the natural heterogeneity of the tumor. Nevertheless, cancer cell lines are adequate models for the research of cancer, and data have demonstrated that the tumor cell lines have a similar response to anticancer drugs when compared to the original tumor [28].

The different human hepatoma cell lines used herein represent various types of tumors with different phenotypes, histhopatologies, protein profiles and clinical outcomes [29,30,31]. These differences limit the search of a unique drug with effective actions on all liver tumors, and encourage the understanding of the distinctive mechanism of action, specific for each tumor. We have found that the aqueous extracts of the *Vismia* and *Piper* genera differentially affected the cell lines. We have found that HepG2 was very sensitive to *V. baccifera*, but not to the *Piper* species; in contrast, the *Piperaceae* induced toxicity to PLC-PRF and SK-HEP-1, but *V. baccifera* did not exert toxic effects. We have seen, therefore, differences in the actions of the extracts depending on the cell type, and this observation is particularly relevant if we consider that not all the patients with liver cancer respond similarly under the same anticancer therapy.

The differential response to the extract may be attributed to differences in genotype and the gene expression profiles in the hepatoma cells, thus leading to the final response. Hepatoma cells secrete high levels of specific proteins, many of which are involved in cell growth and its regulation, and are poorly or not secreted by nontransformed liver cells [32,33,34]. The same proteins have also been detected in clinical specimens from patients’ hepatocarcinomas, thus validating the use of hepatoma cell lines as biological model [34]. One of these markers is alpha-fetoprotein, a protein that is present in 60%–70% of patients with hepatocarcinoma, and represents the only clinical available marker of this liver neoplasm [29]. HepG2 represents a liver cancer cell line with positive alpha-fetoprotein expression, while PLC-PRF and SK-HEP-1 are typical cell lines negative for this protein [28]. As indicated above, these two last cell lines were insensitive to *V. baccifera* toxicity, while the alpha-fetoprotein positive HepG2 cells were highly sensitive to *Vismia*-induced toxicity. In contrast to *V. baccifera*, the *Piperaceae* extracts did not affect the viability of HepG2, but induced toxicity in the two alpha-fetoprotein negative PLC-PRF and SK-HEP-1 lines. Other differences between these two cell types have also been described; PLC-PRF and SK-HEP-1 cells are able to form tumors when injected in nude mice or rats, while no tumors were observed for HepG2 cells [31]. In the present work we describe for the first time a toxic treatment based on aqueous plant extracts that discriminates between these two types of hepatocarcinoma cells that do not share a common protein expression profile.

The anticarcinogenic activity of a potential drug implies the combination of its innocuousness or cytoprotective effect on normal cells and its cytotoxic action on neoplasic cells. The actions of the extracts in normal cells were assayed using primary cultures of rat hepatocytes and the results were compared with those of the rat neoplasic McA-RH7777 cells. This cell line was very sensitive to the extracts-induced toxicity, while the normal counterpart cells were highly resistant, and *P. putumayoense* even protected hepatocytes from cell death. Although data obtained with rat hepatocytes cannot be extrapolated to human normal liver cells, results obtained *in vitro* for several natural compounds point out similar mechanisms of action for the compounds in cells of different origin [35,36]. Nevertheless, the hormetic effects of many compounds should also be considered, as several studies demonstrate opposite effects for the same compound when applied at high or low doses; commonly, there is a stimulatory or beneficial effect at low doses and a toxic effect at high doses [37,38]. Thus, how the plant extracts do regulate and induce the therapeutical effects in cancer needs to be elucidated.

In McA-RH7777 *V. baccifera* and *P. putumayoense* altered cell cycle progression inducing arrest at G2/M phase. These results indicate that these plant extracts probably affected the expression of proteins that regulate transition through the G2 checkpoint, such as cyclin B. Similar effects on cell cycle arrest at G2/M have been reported for theaflavins in human prostate carcinoma and leukemia cells [39,40]. In contrast to *P. putumayoense*, *P. krukoffii* showed no effects on cell cycle progression, suggesting differences in the toxicity mechanism between the two *Piper* species.

We also found that cytotoxicity to human and rat hepatoma cell lines was accompanied by a marked increase of the intracellular ROS production. By contrast, in non-tumor hepatocytes the extracts did not generate ROS and even prevented their basal formation, promoting cell survival. These results suggest that the plant-derived extracts could cause cytotoxicity in malignant human and rat liver cell lines by induction of oxidative stress, and, therefore, by mechanisms independent of their antioxidant actions. Growing evidence suggests that food-derived antioxidants act as chemopreventive agents independent of their free radical scavenging activity. Moreover, antioxidants can become pro-oxidants under specific conditions, such as in the presence of high levels of transition metals. This is the case of naturally occurring compounds, including ascorbic acid and several known anticancer drugs, which act as pro-oxidants in the presence of transition metal ions [41,42,43]. It has been recently described that resveratrol, a phenolic phytochemical present in vegetables and red wine, regulates the expression of proteins involved in the redox balance and apoptosis in SK-HEP-1, suggesting that it causes hepatic cancer cell death by suppressing the expression of antioxidant proteins, and consequently increasing oxidative damage to cells [44]. Our results also showed that the extracts produced significant long-term changes in the activities of antioxidant enzymes, which were reflected by an overall increase in superoxide dismutase activity and a reduction of catalase activity, suggesting the accumulation of hydrogen peroxide. However, we detected no changes in the antioxidant activities at short times (shown in Figure S1); the early oxidative stress induced by the extracts could be responsible for subsequent changes in antioxidant activities, which could contribute to the long-term toxic response.

The early toxic response to the extracts could also be initiated by oxidative stress-independent mechanisms, the intracellular increase of ROS levels being a consequence rather than the cause of cell death. The potential mechanisms involved in the antitumor activities include a) interactions of the components of the extracts with the DNA helix and inhibition of topoisomerases, thus blocking DNA replication and inducing apoptosis; b) inhibition of cytoskeletal proteins which play a key role in cell division; c) perturbations of cell cycle specific proteins, such as cyclins, p27 and p53, blocking proliferation; and d) deregulation (activation/inhibition) of key proteins involved in diverse signaling transduction pathways, such as regulation of cell proliferation and apoptosis (members of the bcl-2 family, phosphatidylinositol-3-kinase, Akt, mitogen activated protein kinases, nuclear factor kappa B or caspases), as has been proposed for natural polyphenols [35]. The role of these signaling pathways in our system are under investigation.

The *in vitro* antitumor activity of plant extracts and infusions, such as tea beverages, has been attributed to their polyphenol components, among them, catechins and epicatechins flavanols being the most abundant [45]. Flavonoids have been extensively reported to exert antitumor actions [46,47,48]. In a preliminary phytochemical screening we found the presence of high levels of flavanols, particularly epicatechin (monomers, dimers and trimers), in *V. baccifera* aqueous infusions [18]. These polyphenols could be responsible for the toxicity induced by the *Hypericaceae* to tumoral cells. Nevertheless, flavanols and other polyphenols of the families flavanones, flavonols, hydroxycinnamic acids, hydroxybenzoic acids, flavones, and coumarins were not detected in *P. krukoffii* and *P. putumayoense* aqueous infusions [18], and here we have shown that the *Piper* species also induced toxicity to tumor cell lines. Other bioactive components of the plant extracts could exert cytotoxicity. In addition, different polyphenolic compounds can also interact synergistically in mediate toxicity and contribute to the final toxic response.

## 5. Conclusions

The present study indicates that aqueous infusions of *V. baccifera*, *P. krukoffii* and *P. putumayoense* induce selective killing of hepatocarcinoma cells, and suggests that ROS could mediate the induced cell toxicities. This system based on the selective responses to plants represents an *in vitro* model to study the mechanisms of action and signaling of cancer cells, and therefore, to better understand cancer biology. In addition, the results described here suggest a need for further research into these plants as promising sources of antitumoral drugs against different types of hepatocarcinoma.

## Supplementary Files

Supplementary File 1

## References

[1] Ferlay J., Shin H.R., Bray F., Forman D., Mathers C., Parkin D.M. (2010). Estimates of worldwide burden of cancer in 2008, GLOBOCAN 2008. Int. J. Cancer.

[2] Parkin D.M., Bray F., Ferlay J., Pisani P. (2005). Global cancer statistics, 2002. C.A. Cancer J. Clin..

[3] Acebo P., Giner D., Calvo P., Blanco-Rivero A., Ortega A.D., Fernández P.L., Roncador G., Fernández-Malavé E., Chamorro M., Cuezva J.M. (2009). Cancer abolishes the tissue type-specific differences in the phenotype of energetic metabolism. Transl. Oncol..

[4] Bensinger S.J., Christofk H.R. (2012). New aspects of the Warburg effect in cancer cell biology. Semin. Cell. Dev. Biol..

[5] Dang C.V. (2012). Links between metabolism and cancer. Genes Dev..

[6] Granchi C., Minutolo F. (2012). Anticancer agents that counteract tumor glycolysis. Chem. Med. Chem..

[7] Mucaj V., Shay J.E., Simon M.C. (2012). Effects of hypoxia and HIFs on cancer metabolism. Int. J. Hematol..

[8] Aninat C., Piton A., Glaise D., Le Charpentier T., Langouët S., Morel F., Guguen-Guillouzo C., Guillouzo A. (2006). Expression of cytochromes P450, conjugating enzymes and nuclear receptors in human hepatoma HepaRG cells. Drug Metab. Dispos..

[9] Donato M.T., Lahoz A., Castell J.V., Gómez-Lechón M.J. (2008). Cell lines: A tool for *in vitro* drug metabolism studies. Curr. Drug Metab..

[10] Vane J.R., Botting R.M. (1998). Anti-inflammatory drugs and their mechanism of action. Inflamm. Res..

[11] Surh Y.J., Chun K.S., Cha H.H., Han S.S., Keum Y.S., Park K.K., Lee S.S. (2001). Molecular mechanisms underlying chemopreventive activities of anti-inflammatory phytochemicals: Down-regulation of CO*_x_*_-2_ and iNOS through suppression of NF-kappa B activation. Mutat. Res..

[12] Kuete V., Nguemeving J.R., Beng V.P., Azebaze A.G.B., Etoa F.X., Meyer M., Bodo B., Nkengfack A.E. (2007). Antimicrobial activity of the methanolic extracts and compounds from *Vismia laurentii* De Wild (Guttiferae). J. Ethnopharmacol..

[13] Osorio E., Arango G.J., Jiménez N., Alzate F., Ruiz G., Gutiérrez D., Paco M.A., Giménez A., Robledo S. (2007). Antiprotozoal and cytotoxic activities *in vitro* of Colombian Annonaceae. J. Ethnopharmacol..

[14] Carvalho M.V., Penido C., Siani A.C., Valente L.M., Henriques M.G. (2006). Investigations on the anti-inflammatory and anti-allergic activities of the leaves of *Uncaria guianensis* (Aublet) J. F. Gmelin. Inflammopharmacology.

[15] Fuller R.W., Westergaard C.K., Collins J.W., Cardellina J.H., Boyd M.R. (1999). Vismiaphenones D-G, new prenylated benzophenones from *Vismia cayennensis*. J. Nat. Prod..

[16] Hussein A.A., Bozzi B., Correa M., Capson T.L., Kursar T.A., Coley P.D., Solis P.N., Gupta M.P. (2003). Bioactive constituents from three Vismia species. J. Nat. Prod..

[17] Lizcano L.J., Bakkali F., Ruiz-Larrea M.B., Ruiz-Sanz J.I. (2010). Antioxidant activity and polyphenol content of Colombian Amazonian plants with medicinal use. Food Chem..

[18] Lizcano L.J., Viloria-Bernal M., Vicente F., Berrueta L.A., Gallo B., Martínez-Cañamero M., Ruiz-Larrea M.B., Ruiz-Sanz J.I. (2012). Lipid oxidation inhibitory effects and phenolic composition of aqueous extracts from medicinal plants of Colombian Amazonia. Int. J. Mol. Sci..

[19] Ruiz-Larrea M.B., Garrido M.J., Lacort M. (1993). Estradiol-induced effects on glutathione metabolism in rat hepatocytes. J. Biochem..

[20] Navarro R., Martínez R., Busnadiego I., Ruiz-Larrea M.B., Ruiz-Sanz J.I. (2006). Doxorubicin-induced MAPK activation in hepatocyte cultures is independent of oxidant damage. Ann. N. Y. Acad. Sci..

[21] Mosmann T. (1983). Rapid colorimetric assay for cellular growth and survival: Application to proliferation and cytotoxicity assays. J. Immunol. Methods.

[22] Bradford M.M. (1976). A rapid and sensitive method for the quantification of microgram quantities of protein utilizing the principle of protein-dye binding. Anal. Biochem..

[23] Flohé L., Günzler W.A. (1984). Assay of glutathione peroxidase. Methods Enzymol..

[24] Aebi H., Packer L. (1984). Catalase *in Vitro*. Methods in Enzymology, Oxygen Radicals in Biological Systems.

[25] López-Torres M., Pérez-Campo R., Rojas C., Cadenas S., Barja G. (1993). Simultaneous induction of sod, glutathione reductase, GSH, and ascorbate in liver and kidney correlates with survival during aging. Free Radic. Biol. Med..

[26] Van Staveren W.C., Solis D.Y., Hebrant A., Detours V., Dumont J.E., Maenhaut C. (2009). Human cancer cell lines: Experimental models for cancer cells *in situ*? For cancer stem cells?. Biochim. Biophys. Acta.

[27] Gazdar A.F., Girard L., Lockwood W.W., Lam W.L., Minna J.D. (2010). Lung cancer cell lines as tools for biomedical discovery and research. JNCI-J. Natl. Cancer Inst..

[28] Finlay G.J., Baguley B.C. (1984). The use of human cancer cell lines as a primary screening system for antineoplastic compounds. Eur. J. Cancer Clin. Oncol..

[29] Fujiyama S., Tanaka M., Maeda S., Ashihara H., Hirata R., Tomita K. (2002). Tumor markers in early diagnosis, follow-up and management of patients with hepatocellular carcinoma. Oncology.

[30] Knowles B.B., Howe C.C., Aden D.P. (2001). Human hepatocellular carcinoma cell lines secrete the major plasma proteins and hepatitis B surface antigen. Science.

[31] Shouval D., Schuger L., Levij I.S., Reid L.M., Neeman Z., Shafritz D.A. (1988). Comparative morphology and tumourigenicity of human hepatocellular carcinoma cell lines in athymic rats and mice. Virchows Arch. A..

[32] Chen C.H., Su K.Y., Tao M.H., Lin S.W., Su Y.H., Tsai Y.C., Cheng K.C., Jeng Y.M., Sheu J.C. (2006). Decreased expressions of hepsin in human hepatocellular carcinomas. Liver Int..

[33] Seow T.K., Liang R.C., Leow C.K., Chung M.C. (2001). Hepatocellular carcinoma: From bedside to proteomics. Proteomics.

[34] Sun Y., Mi W., Cai J., Ying W., Liu F., Lu H., Qiao Y., Jia W., Bi X., Lu N. (2008). Quantitative proteomic signature of liver cancer cells: Tissue transglutaminase 2 could be a novel protein candidate of human hepatocellular carcinoma. J. Proteome Res..

[35] Ramos S. (2008). Cancer chemoprevention and chemotherapy: Dietary polyphenols and signalling pathways. Mol. Nutr. Food Res..

[36] Keum Y.S., Han Y.H., Liew C., Kim J.H., Xu C., Yuan X., Shakarjian M.P., Chong S., Kong A.N. (2006). Induction of heme oxygenase-1 (HO-1) and NAD[P]H: Quinone oxidoreductase 1 (NQO1) by a phenolic antioxidant, butylated hydroxyanisole (BHA) and its metabolite, tert-butylhydroquinone (tBHQ) in primary-cultured human and rat hepatocytes. Pharm. Res..

[37] Zanichelli F., Capasso S., Di Bernardo G., Cipollaro M., Pagnotta E., Cartenì M., Casale F., Iori R., Giordano A., Galderisi U. (2012). Low concentrations of isothiocyanates protect mesenchymal stem cells from oxidative injuries, while high concentrations exacerbate DNA damage. Apoptosis.

[38] Zanichelli F., Capasso S., Cipollaro M., Pagnotta E., Cartenì M., Casale F., Iori R., Galderisi U. (2012). Dose-dependent effects of R-sulforaphane isothiocyanate on the biology of human mesenchymal stem cells, at dietary amounts, it promotes cell proliferation and reduces senescence and apoptosis, while at anti-cancer drug doses, it has a cytotoxic effect. Age.

[39] Prasad S., Kaur J., Roy P., Kalra N., Shukla Y. (2007). Theaflavins induce G2/M arrest by modulating expression of p21waf1/cip1, cdc25c and cyclin B in human prostate carcinoma PC-3 cells. Life Sci..

[40] Ohata M., Koyama Y., Suzuki T., Hayakawa S., Saeki K., Nakamura Y., Isemura M. (2005). Effects of tea constituents on cell cycle progression of human leukemia U937 cells. Biomed. Res..

[41] Ehrenfeld G.M., Shipley J.B., Heimbrook D.C., Sugiyama H., Long E.C., van Boom J.H., van der Marel G.A., Oppenheimer N.J., Hecht S.M. (1987). Copper-dependent cleavage of DNA by bleomycin. Biochemistry.

[42] Hadi S.M., Ullah M.F., Shamim U., Bhatt S.H., Azmi A.S. (2010). Catalytic therapy of cancer by ascorbic acid involves redox cycling of exogenous/endogenous copper ions and generation of reactive oxygen species. Chemotherapy.

[43] Rahman A., Shahabuddin, Hadi S.M., Parish J.H. (1990). Complexes involving quercetin, DNA and Cu(II). Carcinogenesis.

[44] Choi H.Y., Chong S.A., Nam M.J. (2009). Resveratrol induces apoptosis in human SK-HEP-1 hepatic cancer cells. Cancer Genomics Proteomics.

[45] Babich H., Krupka M.E., Nissim H.A., Zuckerbraun H.L. (2005). Differential *in vitro* cytotoxicity of (−)-epicatechin gallate (ECG) to cancer and normal cells from the human oral cavity. Toxicol In Vitro.

[46] Clere N., Faure S., Martinez M.C., Andriantsitohaina R. (2011). Anticancer properties of flavonoids: Roles in various stages of carcinogenesis. Cardiovasc. Hematol. Agents Med. Chem..

[47] Shimizu M., Adachi S., Masuda M., Kozawa O., Moriwaki H. (2011). Cancer chemoprevention with green tea catechins by targeting receptor tyrosine kinases. Mol. Nutr. Food Res..

[48] Yang C.S., Lambert J.D., Sang S. (2009). Antioxidative and anti-carcinogenic activities of tea polyphenols. Arch. Toxicol..

