# Supplementary material for: Piper and Vismia Species from Colombian Amazonia Differentially Affect Cell Proliferation of Hepatocarcinoma Cells
Source: Nutrients. 2014 Dec 30;7(1):179–95. doi: 10.3390/nu7010179 (PMC4303832; doi:10.3390/nu7010179)
Supplement: Supplementary File 1 [file nutrients-07-00179-s001.doc]

Supplementary Information

**Table S1.** Antioxidant enzyme activities in HepG2 cell lines exposed to *V. baccifera*
(75 µg/mL) for 1 h and 3 h.

| **Incubation time** | **SOD** | **GPx** | **Catalase** |
| --- | --- | --- | --- |
| 1 h | 91.5 ± 13.2 | 113.7 ± 5.2 | 100.4 ± 8.5 |
| 3 h | 105.9 ± 13.7 | 102.4 ± 4.7 | 84.4 ± 6.0 |

Activities were measured as U/mg protein (SOD), nmol/min/mg protein (GPx), and µmol/min/mg protein (catalase). Results are expressed as the percentage of the control values (no additions) and are the mean ± SE of *n* = 4–10 experiments. SOD, superoxide dismutase; GPx, glutathione peroxidase.

© 2014 by the authors; licensee MDPI, Basel, Switzerland. This article is an open access article distributed under the terms and conditions of the Creative Commons Attribution license (http://creativecommons.org/licenses/by/4.0/).
